# Design of a Low-Power RFID Sensor System Based on RF Energy Harvesting and Anti-Collision Algorithm

**DOI:** 10.3390/s26031023

**Published:** 2026-02-04

**Authors:** Xin Mao, Xuran Zhu, Jincheng Lei

**Affiliations:** 1 Shien-Ming Wu School of Intelligent Engineering, South China University of Technology, Guangzhou 511442, China; maoxin19233982827@163.com; 2 China Electric Power Research Institute, Beijing 102209, China; zhuxuran@tju.edu.cn

**Keywords:** RF energy harvesting, Dickson charge pump, anti-collision algorithm, embedded system, RFID sensor system

## Abstract

Passive radio frequency identification (RFID) sensing systems integrate wireless energy transfer with information identification. However, conventional passive RFID systems still face three key challenges in practical applications: low RF energy harvesting efficiency, high power consumption of sensor loads, and high complexity of tag anti-collision algorithms. To address these issues, this paper proposes a hardware–software co-optimized RFID sensor system. For hardware, low threshold RF Schottky diodes are selected, and an input inductor is introduced into the voltage multiplier rectifier to boost the signal amplitude, thereby enhancing the radio frequency to direct current (RF-DC) energy conversion efficiency. In terms of loading, a low-power management strategy is implemented for the power supply and control logic of the sensor node to minimize the overall system energy consumption. For algorithmic implementation, a Dual-Threshold Stepped Dynamic Frame Slotted ALOHA (DTS-DFSA) anti-collision algorithm is proposed, which adaptively adjusts the frame length based on the observed collision ratio, eliminating the need for complex tag population estimation. The algorithm features low computational complexity and is well suited for resource constrained embedded platforms. Through simulation validation, we compare the conversion efficiency of the RF energy harvesting circuit before and after improvement, the current of the sensor load in active and idle states, and the performance of the proposed algorithm against the low-complexity DFSA (LC-DFSA). The results show that the maximum conversion efficiency of the improved RF energy harvesting circuit has increased from 60.56% to 68.69%; specifically, the sensor load current drastically drops from approximately 2.0 mA in the active state to around 74 μA in the idle state, validating the efficacy of the proposed power gating strategy, and the proposed DTS-DFSA algorithm outperforms existing low-complexity schemes in both identification efficiency and computational complexity.

## 1. Introduction

With the rapid development of information technology, the Internet of Things (IoT) and wireless sensor networks (WSNs) have expanded quickly, leading to an exponential growth in the number of deployed sensors. Traditional sensor nodes predominantly rely on battery power; however, the limited lifespan, frequent maintenance requirements, and environmental issues caused by discarded batteries make this power supply method unsustainable for large-scale applications [1,2]. In this context, wireless passive sensor systems have become a research hotspot. Among various ambient energy sources, although wind and solar energy [3] have been widely applied, radio frequency (RF) energy has become a promising solution for replacing battery power due to its unique advantages of wide coverage and minimal impact from climate and geography. To further enhance the robustness of wireless power transfer, recent research has also explored advanced modulation techniques to achieve 3D free-positioning, effectively addressing misalignment challenges [4]. Beyond energy acquisition, reliable data transmission is also a core element of wireless passive sensor systems. Particularly in large-scale application scenarios such as logistics warehousing [5] and intelligent inventory [6], wireless passive RFID sensor systems are widely adopted due to their low cost and unique backscatter communication mechanism. In such systems, efficient anti-collision algorithms are crucial to address the channel interference caused by the concurrent communication of massive tags.

As a core component of energy harvesting systems, the RF-DC rectifier dictates the performance of sensor nodes. Existing rectifier designs exhibit significant disparities in performance and can be primarily categorized into CMOS integrated solutions and discrete component-based implementations. In the realm of CMOS technology, recent research has largely focused on mitigating the limitations of diode threshold voltage through complex topologies. For instance, Zheng et al. proposed a Cross-Coupled Differential Drive (CCDD) rectifier operating in the sub-threshold region [7]. By leveraging active threshold compensation techniques, this design achieved a peak power conversion efficiency (PCE) of 70–80% at −20 dBm. However, such high-performance integrated circuits (IC) typically rely on expensive manufacturing processes and stringent production requirements, rendering them less economically viable for cost-sensitive, large-scale RFID tags. Conversely, not all integrated designs guarantee high efficiency; for example, the fully integrated system presented in [8] reached a peak PCE of only 31.77%, which severely restricted its energy harvesting capability.

Alternatively, discrete component-based rectifiers offer lower implementation costs and greater design flexibility, but often entail a compromise between efficiency and form factor. Kumar et al. developed a scalable plug-in rectenna unit that achieved a high PCE of 71% at −10 dBm [9]. Despite its high efficiency, the system operates in the 5.8 GHz frequency band. Compared to 900 MHz, this band faces significantly higher free-space path loss, making it less suitable for long-range backscatter communication. Furthermore, its plug-in 3D structure increases the physical volume, posing challenges for integration into compact planar tags. Another discrete solution designed by Liu et al. operates at 2.4 GHz and integrates a matching network [10]; however, its maximum efficiency is limited to 52.53%, and it requires a relatively high input power of 7 dBm to start up. In summary, existing RF energy harvesting solutions struggle to achieve high power conversion efficiency in the Ultra High Frequency (UHF) band under low-cost constraints. Developing a rectifier solution that effectively balances high performance, low manufacturing cost, and an appropriate communication frequency remains a critical challenge in meeting the practical application requirements of large-scale passive IoT nodes.

In the pursuit of overcoming RF energy harvesting bottlenecks, anti-collision algorithms serve as the core mechanism for ensuring communication reliability, making their performance optimization crucial. To enhance system throughput, a series of state-of-the-art solutions have pushed identification efficiency toward theoretical limits. For instance, Li et al. proposed the Deep-Gated Multistage Frame Slotted ALOHA (D-G-MFSA) [11], which integrated Long Short-Term Memory (LSTM) networks into the Dynamic Frame Slotted ALOHA (DFSA) framework to predict tag populations, achieving a peak throughput of 36.8%. Similarly, Xue et al. employed Compressed Sensing and Orthogonal Matching Pursuit (CS-OMP) to separate collided signals in the frequency domain, effectively breaking the Nyquist sampling limit [12]. However, in-depth comparative analysis reveals that these high-performance algorithms rely heavily on intensive neural inference or complex matrix iterative operations. As demonstrated by the quantitative analysis in Section 3.3 of this study, such computational intensity necessitates substantial floating-point arithmetic and memory resources, often exceeding the real-time processing capabilities of low-cost, resource-constrained Microcontroller Unit (MCU).

Although deterministic tree algorithms, represented by the Dual Response Collision Tree (DRCT) proposed by Zhou et al. [13], can achieve an exceptionally high channel utilization of 84.8%, such protocols rely critically on precise state machine management and frequent prefix matching. In dynamic environments involving large-scale tag populations, maintaining multi-level depth counters leads to a significant increase in firmware complexity and introduces identification latency, posing substantial challenges for integration into standard RFID sensing platforms. To mitigate algorithmic overhead, early heuristic strategies such as Threshold DFSA (THDFSA) [14] and LC-DFSA [15] attempted to simplify the estimation process. However, the efficiency of THDFSA is heavily constrained by the accuracy of the initial tag estimation, making it prone to cumulative errors in fluctuating environments. Meanwhile, although LC-DFSA reduces complexity through pre-stored split points, it lacks fine-grained adjustment mechanisms to cope with intermittent signal conditions. Despite these technological breakthroughs, a critical research gap remains: how to find the optimal equilibrium between identification efficiency and computational overhead. Most existing advanced solutions tend to pursue extreme throughput at the expense of excessive hardware resources, which is far from ideal for RFID sensing nodes that prioritize low cost and low power consumption.

Targeting these challenges, this paper proposes a hardware–software co-optimized wireless battery-free RFID sensor system. The main contributions are summarized as follows:

1. An improved Dickson charge-pump rectifier is proposed. By integrating a series inductor and optimizing the impedance matching design, the RF energy harvesting efficiency is significantly enhanced.
2. A low-power management strategy is introduced. Utilizing dynamic task scheduling and module-level power control, this strategy minimizes system energy consumption while ensuring reliable operation under intermittent energy supply.
3. A DTS-DFSA algorithm tailored for resource-constrained embedded platforms is proposed. This algorithm innovatively uses the collision ratio as a single feedback metric, eliminating the need for complex tag population estimation. Consequently, it significantly reduces computational complexity and hardware overhead while maintaining high tag identification efficiency.

## 2. Methodology

### 2.1. System Architecture

**Figure 1:**
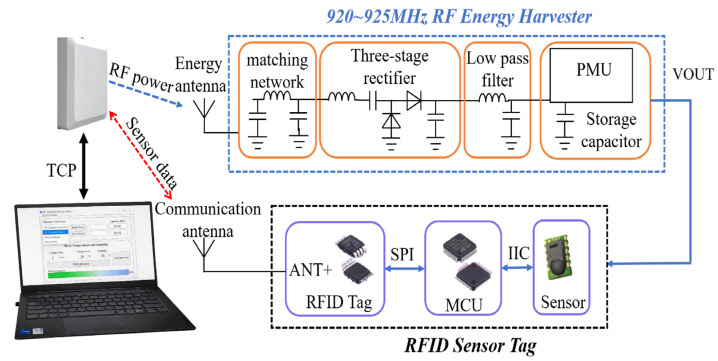
Schematic of an optimized RF energy powered RFID sensor system incorporating an anti-collision algorithm.

As illustrated in Figure 1, the proposed passive RFID sensor system consists of three main modules: an RFID reader, an RF energy harvesting module, and a MCU based temperature and humidity sensor. These modules collaboratively form an energy autonomous, low-power wireless sensing system. The central design concept focuses on the co-optimization of the RF energy harvesting circuit, low-power sensor design, and anti-collision protocol, which jointly enhance energy utilization efficiency and multi-tag identification performance through integrated optimization at both the hardware and algorithmic levels.

The RFID reader serves as both the RF energy source and the central controller for communication and coordination. During different operational stages, the reader performs distinct functions. In the charging phase, it provides energy to the tag. In the wake-up phase, it transmits control commands to activate the MCU. And in the communication phase, it exchanges data with the tag through Query and acknowledgment (ACK) commands.

On the tag side, the RF energy harvesting circuit is responsible for rectifying and voltage multiplying the input signal, while the energy management module regulates and stores the harvested energy to provide a stable power supply for the MCU and the sensor. The MCU drives the sensor to perform environmental data acquisition and writes the measured data into a designated memory area of the tag for subsequent reading by the reader.

The system workflow is coordinated by a host computer which communicates with the reader via the Transmission Control Protocol (TCP). The host computer first sets the charging duration, during which the reader continuously emits RF energy without waking up the MCU. Once the charging time elapses, the host computer instructs the reader to send a wake-up command, prompting the MCU to enter the working state. The MCU drives the temperature and humidity sensor via the Inter-Integrated Circuit (I2C) interface for measurements, and transmits the results to a specific memory region of the tag through the Serial Peripheral Interface (SPI) interface. The reader then retrieves the data to obtain temperature and humidity information. After completing the measurements, the MCU shuts off the sensor power via coordinated software and hardware control and enters a low-power sleep mode to minimize energy consumption.

The overall operational cycle of the system can be summarized as “charging-wakeup-operation-sleep”. By adopting this periodic self-powered mechanism, the system achieves low-power, sustainable performance.

**Figure 2:**
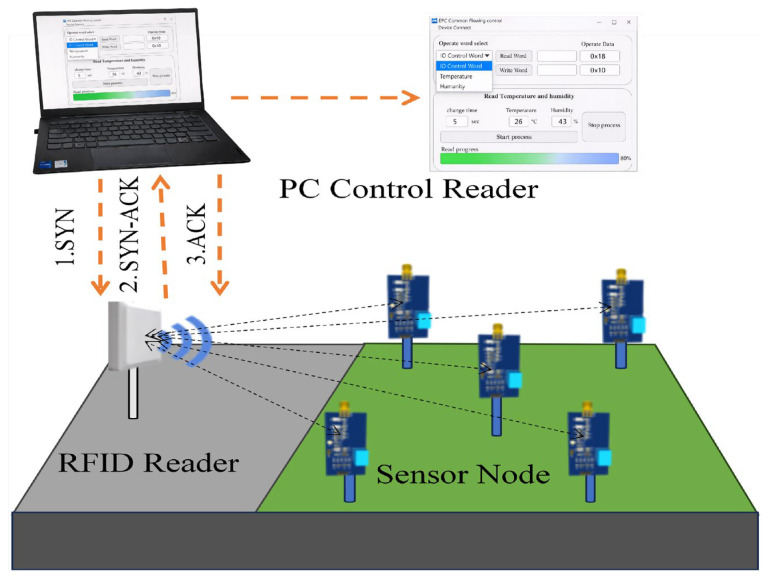
Operation scenario diagram of the proposed passive RFID sensor system.

As illustrated in Figure 2, the proposed system architecture offers versatility and scalability, enabling flexible deployment across diverse environments. It is suitable for indoor applications such as laboratory monitoring, warehouse management, and temperature–humidity sensing, and can also be extended to outdoor scenarios including agricultural monitoring, environmental sensing, and structural health inspection. The entire system operates without any battery, featuring low power consumption, maintenance-free operation, and high reliability, thereby providing an efficient and sustainable solution for next-generation passive IoT sensing networks.

### 2.2. RF Energy Harvesting

As shown in Figure 1, the proposed RF energy-harvesting circuit operates in the 920–925 MHz frequency band and is composed of an antenna, a 50 Ω feeder, an impedance matching network, an improved three-stage Dickson voltage multiplier, a low-pass filter, and an energy management module.

The UHF signal received by the antenna is transmitted through the 50 Ω feeder and further optimized by the matching network to maximize power transfer efficiency, ensuring that the RF energy is effectively coupled into the rectifier. The improved three-stage Dickson structure performs voltage multiplication and rectification of the incoming RF signal into DC power, effectively reducing diode threshold losses and enhancing RF-DC conversion efficiency. The subsequent low-pass filter suppresses harmonics and voltage ripples, providing a stable DC output, while the energy management module regulates voltage and manages energy storage, ensuring a stable and reliable power supply for the MCU and sensor.

The following section provides a detailed analysis of voltage multiplier rectifier topologies, the rectifying diode selection, and improvements in the Dickson voltage multiplier topology.

#### 2.2.1. Voltage Multiplier Rectifier Topologies

In RF energy harvesting systems, the voltage multiplier rectifier is the key stage that determines overall RF-DC conversion efficiency. The two most widely used topologies are the Villard and Dickson multiplier [15].

The Villard topology achieves voltage multiplication by alternately charging and discharging the coupling capacitors during each half-cycle, effectively boosting the output voltage under low-to-moderate input power conditions. The Dickson topology operates based on an alternating-phase charge-pump mechanism, in which the coupling capacitors and diodes conduct synchronously in alternating phases, enabling efficient charge transfer and stable voltage retention.

Since the Dickson topology adopts an alternating-phase charge and discharge mechanism between adjacent stages, it can utilize the input signal energy more efficiently and improve charge transfer performance. In contrast, the Villard topology employs a single phase charge–discharge mode, which suffers from insufficient conduction time at high frequencies, thereby limiting energy transfer. The alternating-phase design of the Dickson topology effectively suppresses reverse charging across the capacitors and reduces dynamic energy losses. Moreover, its larger effective conduction angle enables the output voltage to remain stable under high-frequency conditions, whereas the Villard topology exhibits significant voltage attenuation due to parasitic capacitance effects.

**Figure 3:**
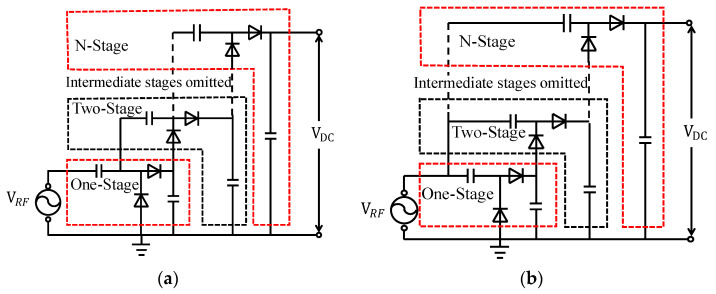
Circuit topologies of RF energy harvesting rectifiers: (**a**) Villard voltage multiplier; (**b**) Dickson charge-pump rectifier. The dashed lines indicate omitted intermediate stages, and the red and black outlines are used only to highlight the first-stage structure for clarity, without any specific physical meaning.

To further verify the performance differences between the two circuits, simulations were conducted on the Keysight Advanced Design System (ADS) 2022 Update 2 (64-bit Simulations) platform under identical component parameters and operating conditions, based on the topologies shown in Figure 3.

#### 2.2.2. Diode Selection Criteria

In rectifier voltage multiplying circuits, the performance of diodes directly determines the RF-DC conversion efficiency. Besides conventional Schottky diodes, CCDD rectifiers have been proposed in recent studies to further suppress the threshold effect. However, CMOS-based CCDD rectifiers suffer from high design complexity and strong process dependence. As a result, low-threshold Schottky diodes remain the most widely used choice in RFID and Internet of Things energy-harvesting systems [16].

In the device selection process of this study, the following key parameters are emphasized:

- Forward voltage ${(V}_{f})$: Determine the diode’s conduction capability under low-power signals. A lower $V_{f}$ reduces the turn-on barrier.
- Reverse leakage current ($I_{r}$): Reflect the energy loss in the cut-off state. Excessive leakage current degrades overall system efficiency.
- Junction capacitance ($C_{t}$): At high operating frequencies, junction capacitance introduces signal attenuation and delay. A suitably low $C_{t}$ improves rectification efficiency and high-frequency response.

Based on the above selection criteria, a comparative analysis of several commonly used RF diodes was conducted, and the results are presented in Table 1. The SMS7630 Schottky diode (Skyworks Solutions, Inc., Irvine, CA, USA) exhibits a forward voltage drop ranging from 135 mV to 240 mV at 1 mA, which is overall lower than that of most comparable devices, effectively reducing threshold-related losses. In terms of junction capacitance, the SMS7630 has a value of approximately 0.3 pF, which is significantly smaller than that of the HSMS-282x and BAT17-04W diodes but slightly higher than that of the SMS7621. This moderate capacitance is advantageous for achieving good rectification performance in the UHF band. It should be noted that the table does not provide the reverse leakage current data (Ir@2 V) for the SMS7630, and thus a direct comparison of this parameter cannot be made. Overall, the SMS7630 combines a low forward voltage drop with a small junction capacitance under low-power conditions, demonstrating excellent rectification and energy conversion characteristics. Therefore, it was selected as the core rectifying device in this study.

#### 2.2.3. Inductive Resonance Dickson Rectifier with Low-Threshold Diodes

**Figure 4:**
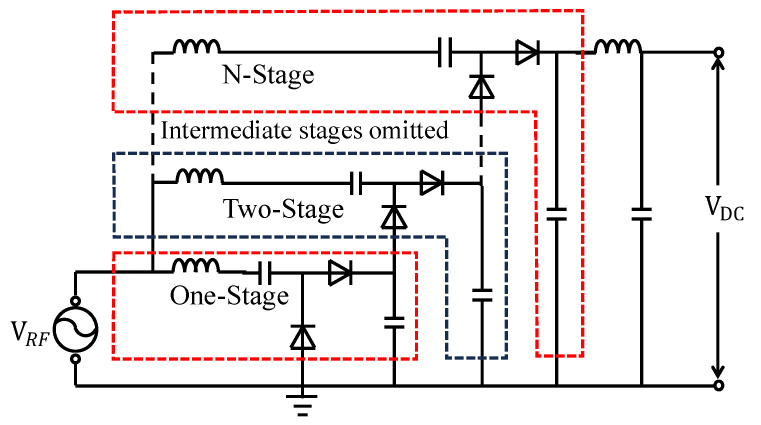
Circuit topology of the improved Dickson charge-pump rectifier. The dashed line indicates that intermediate stages are omitted for simplicity, and the colored outlines are used only to highlight different rectifier stages for visual clarity, without any specific physical or electrical meaning.

To improve rectification efficiency, in addition to adopting low-threshold Schottky diodes, a resonant network was introduced to enhance the RF voltage swing within the target frequency band. This approach helps to overcome the diode threshold voltage, widen the conduction angle, and extend the effective charging duration, thereby further improving the energy conversion efficiency. The circuit principle is illustrated in Figure 4. Furthermore, a low-pass filter was incorporated at the output stage of the Dickson voltage multiplier to suppress high-frequency components and smooth the output voltage. This provides a more stable signal to the energy management unit, enhances overall conversion efficiency, and protects the subsequent circuitry.

To gain deeper insight into the superior performance of the Dickson rectifier and to clarify the intrinsic limitations of RF-DC conversion efficiency, a theoretical analysis was carried out. First, the input impedance and reflection characteristics of the rectifier were investigated. Subsequently, the influence of the diode threshold voltage and conduction angle on charge-transfer efficiency was analyzed, providing a theoretical basis for understanding the performance boundaries of Dickson-based rectifiers.

Due to the combined effects of the diode junction capacitance and the interstage coupling capacitance, the input impedance of the voltage-multiplier rectifier generally exhibits a capacitive characteristic within the operating frequency band. Without impedance matching, the equivalent load impedance $Z_{in}$ observed at the source can be approximately expressed as Equation (1):

(1) $$Z_{in}\approx \frac{1}{j\omega C_{in}}∥\;R_{eq}$$

(2) $$C_{in}={C_{j}+C}_{c}+C_{par}$$

(3) ω=2πf

where ω denotes the angular frequency, which is related to the operating frequency f as shown in Equation (3), $C_{in}$ represents the input capacitance, $C_{j}$ is the diode junction capacitance, $C_{c}$ is the interstage coupling capacitance, and $C_{par}$ denotes the parasitic plate capacitance (Equation (2)), and $R_{eq}$ represents the equivalent load resistance.

Under this condition, the reflection coefficient Γ of the system can be expressed as:

(4) $$\Gamma =\;\frac{Z_{in}-Z_{0}}{Z_{in}+Z_{0}}$$

where $Z_{0}$ denotes the characteristic impedance of the transmission line, which is set to 50 Ω in this work.

The fraction of incident power effectively absorbed by the rectifier can be expressed as Equation (5):

(5) $$1-{\left|\Gamma \right|}^{2}$$

To maximize the incident power, i.e., to minimize the reflection coefficient Γ, an inductive component can be introduced at the input to form an impedance-matching network, satisfying Equation (6). In this case, the input impedance $Z_{in}$ will approach a real value, and the reflection coefficient *Γ* will be minimized, thereby maximizing the incident power absorption ratio.

(6) $$\omega L=\frac{1}{\omega C_{in}}⟹\omega^{2}LC_{in}\approx 1$$

Here, *L* represents the value of the introduced inductance.

This result indicates that, when resonance matching is achieved between the input inductor and capacitor, the inductance value is inversely proportional to the input capacitance. Such a relationship provides a theoretical basis for impedance-matching design and establishes a foundation for improving the power transfer efficiency of the rectifier within the operating frequency band.

The conduction angle of the diode is determined by the amplitude of the input signal. A diode conducts only when the instantaneous voltage exceeds the threshold voltage. Assuming that the input signal Vin can be approximated as:

(7) $$V\left(t\right)=V_{RF}\;Sin(\omega t)$$

where $V_{RF}$ denotes the peak amplitude of the RF input voltage, t represents time with the unit in second. The conduction angle $\theta_{cond}$ within a single cycle can be expressed as:

(8) $$\theta_{cond}\approx 2\arccos(\frac{V_{\mathrm{th}}}{V_{RF}})$$

where $V_{\mathrm{th}}$ denotes the diode threshold voltage.

The output voltage $V_{out}$ of the voltage multiplier rectifier can be approximated as:

(9) $$V_{out}\approx n\left(V_{RF}-V_{th}\right)$$

where *n* denotes the number of multiplier stages. The definition of energy conversion efficiency η is given as follows:

(10) $$\eta =\;\frac{P_{out}}{P_{in}}=\frac{V_{out}I_{out}}{P_{in}}\times 100\%$$

where $P_{out}$ and $P_{in}$ represent the output and input power of the voltage multiplier rectifier circuit. $V_{out}$ and $I_{out}$ represent the output voltage and output current of the voltage multiplier rectifier circuit, respectively, and $P_{out}$ is proportional to the product of $V_{out}$ and $I_{out}$.

Therefore, under the resonant condition with an added inductor, it can not only reduce reflection loss and increase signal amplitude, but also effectively improve the conduction duty cycle of the diode, thereby enhancing the overall energy conversion efficiency.

Based on this principle, this paper conducts a simulation analysis on the improved Dickson voltage multiplier rectifier to verify its enhanced RF-DC PCE.

#### 2.2.4. Circuit Simulation

To validate the feasibility of the improved topology proposed based on the aforementioned theory, this study, oriented toward the practical application requirements of RF energy harvesting circuits, performed circuit-level simulation verification using ADS. In the simulations, the SMS7630 was employed as the rectifier diode. By invoking its S-parameter model, deviations between the ideal diode model and its actual characteristics could be eliminated, thereby ensuring simulation accuracy. For the passive components, the GRM series for capacitors and the LQW series for inductors (both from Murata Manufacturing Co., Ltd., Kyoto, Japan) [10] provide high quality factors and low parasitic parameters, which can reduce passive losses in practical applications. During the simulation, the S-parameter models of these passive components were also incorporated to guarantee the reliability and authenticity of the simulation. Both the selection analysis of the two typical voltage multiplier topologies that are Dickson and Villard and the determination of the optimal number of stages, as discussed in the preceding section, were supported by the quantitative data obtained after modifications in this simulation.

**Figure 5:**
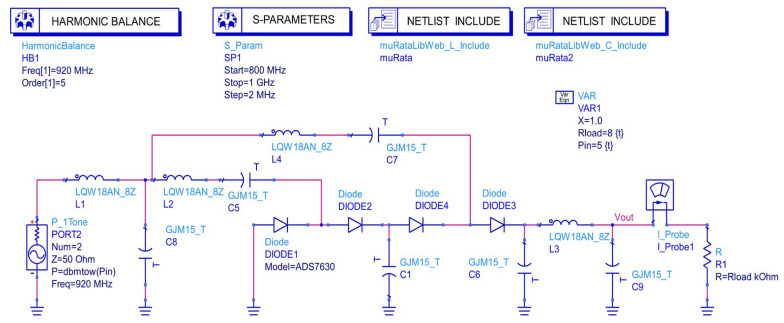
Schematic of the ADS simulation on the designed RF energy harvesting circuit.

The simulation was carried out at a fixed frequency of 920 MHz, which was consistent with the mainstream frequency band commonly used in RF energy harvesting applications. The input power was linearly swept from −12 dBm to +5 dBm with a step size of 0.5 dBm, covering typical scenarios ranging from weak to moderately strong RF signals. Three commonly used load resistance values of 10 kΩ, 20 kΩ, and 30 kΩ were selected. Based on real-time monitoring of PCE, the optimal load resistance that maximizes circuit efficiency was determined. The corresponding simulation schematic and parameter settings are shown in Figure 5.

On this basis, the simulation setup was further modified to conduct a comparative analysis between the Dickson and Villard voltage multiplier topologies. The PCE performance of Dickson rectifiers with different stage numbers was also evaluated to investigate the influence of stage variation on overall efficiency. In addition, the improved Dickson rectifier was compared with the conventional structure in terms of PCE, as well as the input-output voltage characteristics before and after optimization. The results confirm the superior energy conversion efficiency and voltage boosting capability of the improved Dickson topology.

To validate the proposed topology and guide hardware implementation, circuit-level simulations were performed in Keysight ADS 2022. The study (i) compared the RF-DC conversion efficiency of Dickson and Villard rectifiers and determined the optimum number of stages; (ii) quantified the effects of diode type and input inductance/capacitance on efficiency for parameter optimization; and (iii) predicted the attainable efficiency over the target input-power and frequency ranges to inform Printed Circuit Board (PCB) impedance matching.

### 2.3. MCU Runtime Strategy

In the operation of sensor nodes, temperature measurement is performed only when requested by the host computer. However, in most applications, such requests occur infrequently, and the measurement duration is relatively short, leading to significant energy waste during prolonged idle periods of the node [19].

To address this issue, the system employs low-power communication chips and implements a working strategy based on alternating listening and sleep modes. During non-operational periods, the node automatically enters a sleep state instead of remaining idle, thereby effectively reducing energy consumption and extending system lifetime.

Furthermore, targeted power optimization was applied to the system load to achieve minimal power consumption and enhance overall energy efficiency.

**Figure 6:**
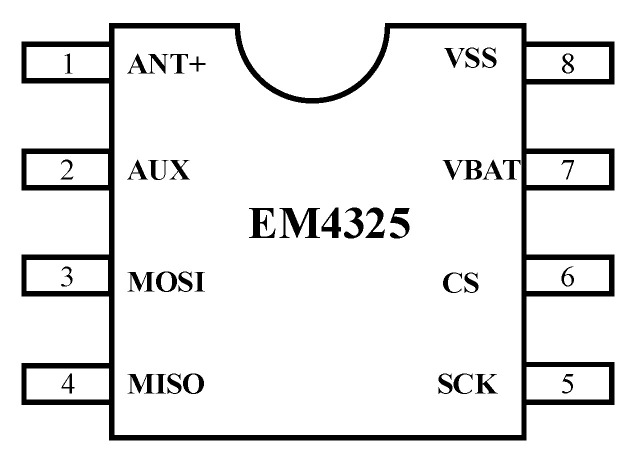
Pin configuration diagram of the EM4325 RFID chip, where the numbers denote the corresponding pin numbers.

In this system, the auxiliary pin (AUX) as defined in Figure 6 of the RFID chip EM4325 (EM Microelectronic, Marin, La Tène, Switzerland) is configured as a wake-up control signal for the MCU. Once the AUX function is enabled, the EM4325 outputs a high-level pulse on the AUX pin as soon as it detects a valid interrogation signal from the reader, thereby triggering the MCU to wake from its low-power state.

During the power-up phase, all unused MCU Input/Output (I/O) pins are first configured as analog inputs to minimize leakage currents caused by floating or undefined logic levels. When communication with the SHT10 (Sensirion AG, Stäfa, Switzerland) is required, only the corresponding pins are temporarily reconfigured to the I2C mode; after the measurement is completed, these pins are immediately restored to the analog input mode. Similarly, during communication with the EM4325, the relevant pins are switched to the SPI mode only for the data transfer period and then reverted to the analog input configuration [20]. This dynamic pin reconfiguration strategy effectively suppresses static power consumption in non-operational states.

**Figure 7:**
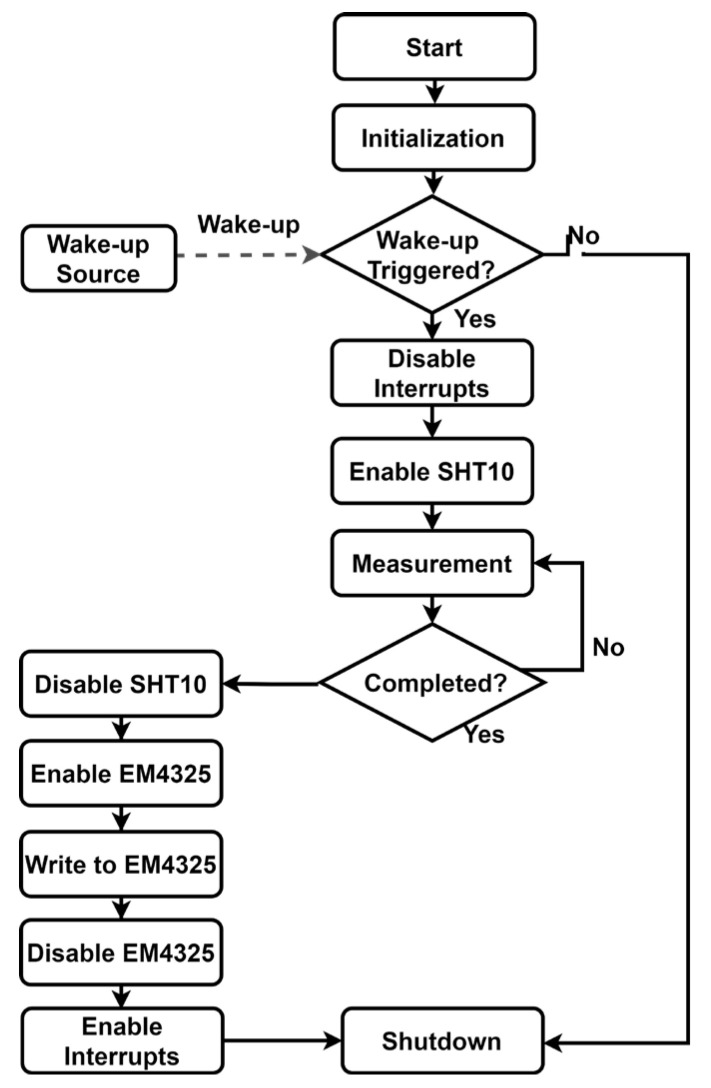
Flowchart of the MCU runtime strategy in the passive RFID sensor tag.

When the reader initiates a read request, continuous RF energy is transmitted to power the tag. After the charging window, the host activates the AUX output of the EM4325 to wake up the MCU. Once awakened, the MCU immediately executes a dynamic power management sequence for peripheral devices, as shown in Figure 7.

In the sensor data acquisition phase, the MCU enables the power supply of the SHT10 through a MOSFET switch. After the temperature and humidity measurement is completed and the data are acquired, the MCU promptly disconnects the sensor power, thereby confining the energy consumption of the SHT10 to a short measurement window only.

In the data transmission and sleep phase, after data preprocessing, the MCU enables the power interface of the EM4325 and writes the valid data into its internal memory via the SPI bus. Once the data transfer is completed, the MCU turns off the power of the EM4325 through a MOSFET and subsequently enters the Shutdown mode to wait for the next wake-up event.

By jointly applying these three strategies at the pin configuration, peripheral power control, and system sleep levels, the proposed design ensures that energy is consumed only when strictly necessary. This coordinated power management significantly reduces standby power consumption and improves energy utilization efficiency, making the system well suited for ultra-low-power passive or semi-passive sensing tag applications.

### 2.4. Anti-Collision Algorithm

**Figure 8:**
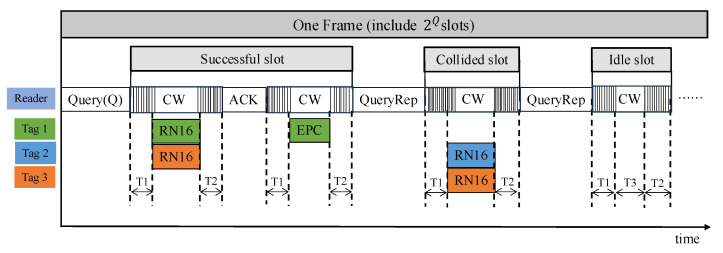
Schematic of Frame Slot States in the Anti-collision algorithm. The ellipsis indicates that subsequent frames follow the same slot structure as the preceding ones and are omitted for simplicity.

In RFID systems, various anti-collision algorithms have been developed to address signal conflicts caused by simultaneous tag responses. Among them, Reference [21] provides a systematic comparison and detailed analysis of four fundamental anti-collision algorithms, offering a theoretical foundation for subsequent algorithmic improvements and optimizations. In the DFSA protocol of an RFID system, the interaction between the reader and tags is carried out through discrete time slots, as illustrated in Figure 8. The entire identification process is initiated by the reader through the Query (Q), which contains the parameter Q determining the frame length. Specifically, the frame consists of $2^{Q}$ time slots, where Q ranges from 0 to 15. For example, when Q = 4, the frame length is 16 slots [21,22].

Upon receiving the Query command, each tag randomly generates an integer within the range [0, $2^{Q}-1$], loads it into its slot counter, and then enters the arbitration state. In the arbitration state, tags continuously monitor QueryRep commands sent by the reader. Each time a QueryRep command is received, the tag decrements its slot counter by one. When the counter reaches zero, the tag switches to the reply state and backscatters a 16-bit random number (RN16) as its response signal.

During each time slot, three possible outcomes can occur: a successful slot, a collided slot, or an idle slot. A slot is considered successful when the reader successfully demodulates and decodes a unique RN16. In this case, the reader sends an ACK containing that RN16. The tag, upon receiving the ACK, verifies whether the included RN16 matches its own transmitted one. If the two match, the tag enters the confirmation state and transmits its complete identification information, including the Protocol Control Word, Electronic Product Code (EPC), and the 16-bit Cyclic Redundancy Check (CRC-16). After completing data transmission, the tag transitions into the silent state, withdrawing from subsequent inventory rounds.

The DTS-DFSA with Dynamic Frame Size Adjustment is a simplified anti-collision control method based on the collision ratio (*CR*). The main idea is to monitor the proportion of collided slots in each frame in real time and dynamically adjust the frame length accordingly. This approach achieves adaptive frame length optimization while significantly reducing computational complexity.

A dual-threshold mechanism is adopted: when the *CR* exceeds the upper threshold, severe collisions occur, and the frame length must be enlarged to disperse tag responses; when the *CR* is below the lower threshold, channel utilization is insufficient, and the frame length should be shortened to increase throughput; when the *CR* lies within the stable region, the frame length remains unchanged. This mechanism ensures system stability while lowering computational overhead, making it particularly suitable for low-power embedded implementations.

The collision ratio is defined as [23]:

(11) $$CR\;=\;\frac{N_{collision}}{N_{total}}$$

where $N_{collision}$ denotes the number of collided slots within the frame, and $N_{total}$ represents the total number of slots in the current frame.

The dual-threshold values are set as:

(12) CR = 0.7    CR = 0.3

The corresponding adjustment strategies for frame length based on *CR* values are summarized in Table 2.

## 3. Results and Discussion

### 3.1. Simulation of RF Energy Harvesting Circuit

This section presents a systematic evaluation and comparative study of RF energy-harvesting circuits on the aforementioned simulation platform. First, we compare the performance differences between the canonical Dickson and Villard voltage multiplier rectifier topologies. Second, we assess how the output capability and power-conversion efficiency evolve with the number of stages in Dickson multiplier. Finally, we characterize key parameters of the improved Dickson topology and benchmark its RF-DC conversion efficiency and stability against the baseline design to quantify the performance gains.

#### 3.1.1. Performance Evaluation of Voltage Multiplier Rectifier Circuits

**Figure 9:**
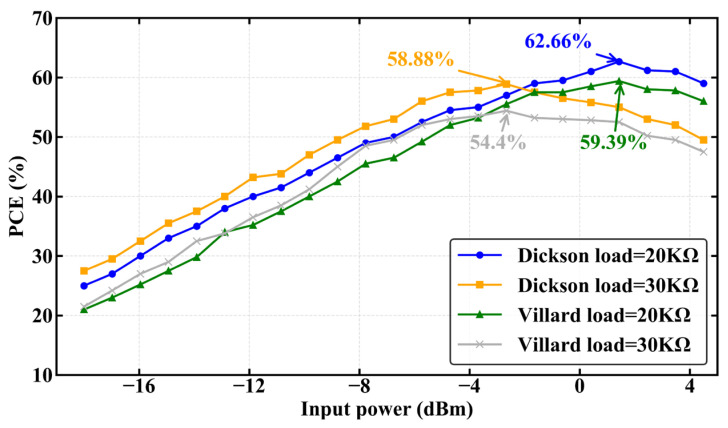
Comparison of RF-DC PCE between the Dickson and Villard voltage multiplier rectifiers under different load conditions.

As shown in Figure 9, within the target input power range of −12 dBm to +5 dBm, the Dickson voltage multiplier achieves a peak efficiency of approximately 65% when the load resistance is $R_{L}$ = 30 kΩ, whereas the peak efficiency of the Villard multiplier remains below 55% across the same range. These results clearly indicate that, within the specified input power range, the Dickson voltage multiplier exhibits significantly higher efficiency compared to the Villard topology.

**Figure 10:**
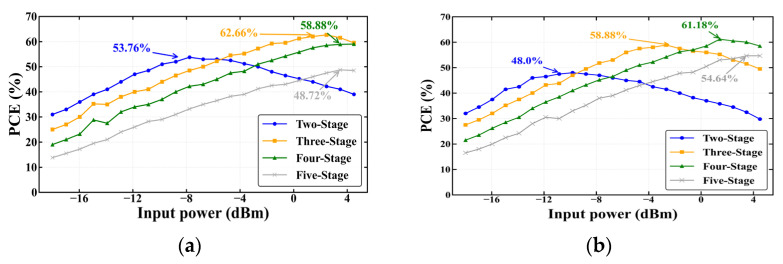
PCE versus input power for Dickson voltage multiplier with different stage numbers operating at 920–925 MHz: (**a**) Load = 20 kΩ; (**b**) Load = 30 kΩ.

As illustrated in Figure 10, within the input power range of −8 dBm to +3 dBm, the three-stage Dickson voltage multiplier exhibits the highest PCE, achieving a peak efficiency of approximately 60–65%. In contrast, the two-stage Dickson configuration, owing to its lower effective threshold voltage, demonstrates a slight advantage only when the input power is below −8 dBm. Although the four-stage and five-stage Dickson structures can theoretically provide higher voltage multiplication, their overall efficiency is constrained by the cumulative effects of diode forward voltage drops and parasitic capacitances, which significantly compress the conduction angle. As a result, the efficiency crossover occasionally occurs only at higher input power levels.

Considering the preceding comparison with the Villard topology, the three-stage Dickson structure achieves an optimal trade-off among conversion efficiency, impedance controllability, and implementation complexity. Therefore, all subsequent circuit optimizations and experimental validations in this study are based on the three-stage Dickson voltage multiplier.

#### 3.1.2. Simulation of Inductive-Resonance Dickson Rectifier with Low-Threshold Diodes

**Figure 11:**
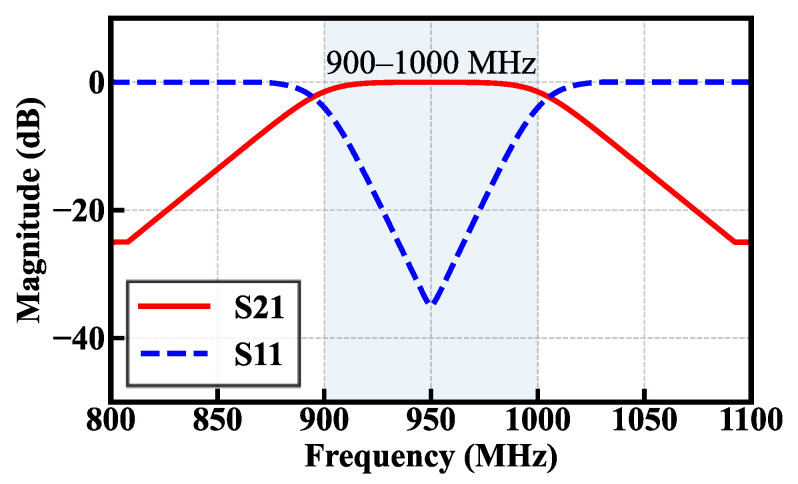
S-parameter response of the improved Dickson voltage multiplier rectifier from 800 to 1100 MHz.

An L-type impedance matching network is adopted in this study owing to its simple structure, low component count, and minimal loss, making it highly suitable for narrowband RF energy harvesting applications in the 920–925 MHz range. As shown in Figure 11, the reflection coefficient curve indicates that good impedance matching is achieved between the antenna and the rectifier circuit within the target frequency band, resulting in a significant reduction in signal reflection. Consequently, more incident RF energy can be effectively transferred to the RF-DC conversion circuit, thereby improving the overall energy harvesting efficiency.

**Figure 12:**
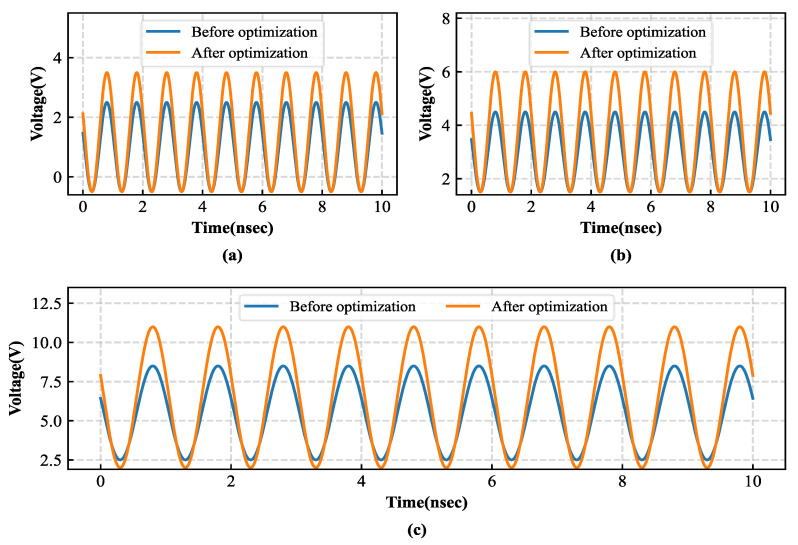
Comparison of input-node voltage waveforms of the Dickson voltage multiplier before and after optimization: (**a**) one-stage; (**b**) two-stage; (**c**) three-stage.

Figure 12 compares the input voltage amplitudes of each stage of the Dickson voltage multiplier rectifier before and after introducing an input inductor. The results show that the circuit with the added inductor exhibits a noticeable increase in input voltage amplitude at each stage, and the improvement becomes more pronounced as the number of stages increases. This result demonstrates that the introduction of the input inductor effectively raises the diode input voltage, thereby mitigating the limitation imposed by the diode threshold voltage and providing a solid foundation for improving the overall RF-DC conversion efficiency of the circuit.

**Figure 13:**
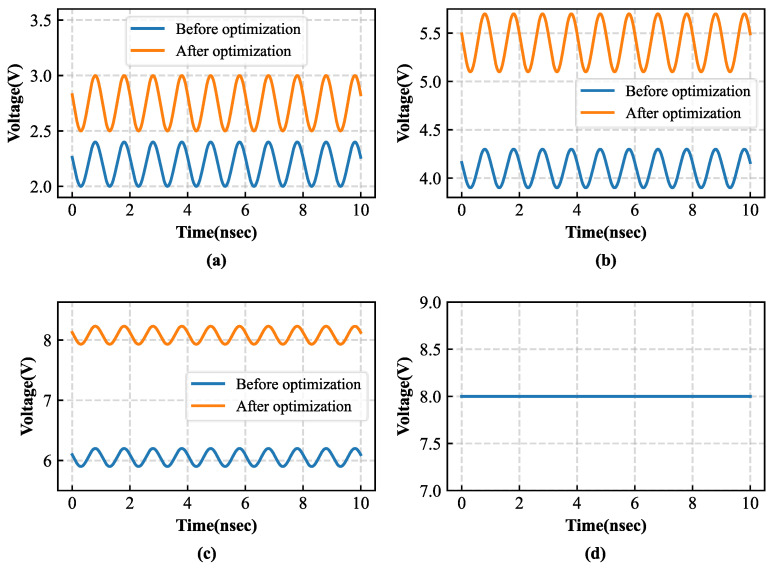
Comparison of output-node voltage waveforms of the Dickson voltage multiplier before and after optimization: (**a**) one-stage output; (**b**) two-stage output; (**c**) three-stage output; (**d**) filtered output.

Figure 13 compares the output voltage amplitudes of each stage of the Dickson voltage multiplier rectifier before and after introducing an input inductor and adopting the SMS7630 RF Schottky diode. The results show that the optimized circuit exhibits a significant increase in output voltage amplitude at each stage, with the improvement becoming more pronounced as the number of stages increases. The voltage enhancement indicates that the energy transfer efficiency of each rectifying stage is improved, thereby providing a higher available voltage for subsequent energy storage and conversion processes. Figure 13d presents the final output voltage waveform of the improved circuit after low-pass filtering. The output voltage is smoother and more stable, demonstrating that the optimized design achieves superior voltage gain and energy transfer performance. This improvement enables the energy management module to receive a more stable and reliable input, further enhancing the overall operational efficiency and stability of the system.

**Figure 14:**
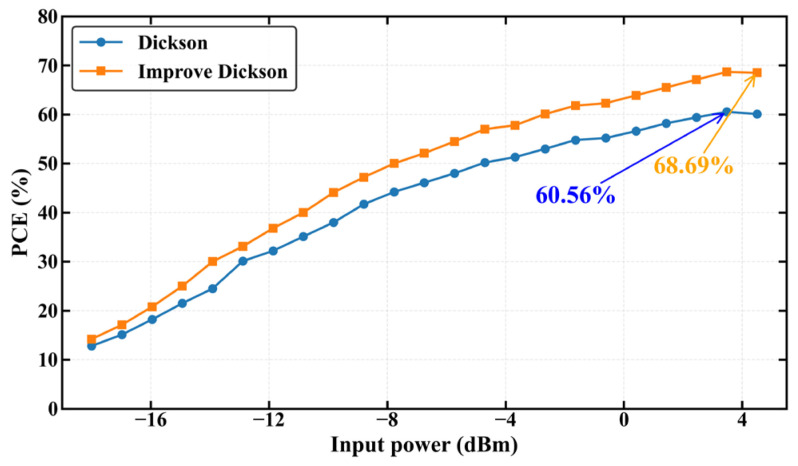
Plots of RF-DC conversion efficiency versus input power with and without an input inductor.

As shown in Figure 14, the RF-DC conversion efficiency of the Dickson voltage multiplier rectifier was compared before and after the introduction of the input inductor and the adoption of the SMS7630 RF Schottky diode. The results reveal that, within the medium-to-high input power range, the improved circuit exhibits a significant enhancement in energy conversion performance, with the peak efficiency increasing from 60.56% to 68.69%. This trend is consistent with the observed increase in output voltage, further confirming that the proposed optimization mechanism effectively improves the impedance matching and energy transfer efficiency. The overall efficiency curve shifts upward, demonstrating that the optimized Dickson voltage multiplier rectifier achieves a synergistic improvement in both voltage gain and conversion efficiency, providing strong support for the design of high-efficiency RF energy-harvesting systems.

#### 3.1.3. Experimental Validation of RF Energy Harvesting Circuit

To validate the practical performance of the proposed RF energy harvesting circuit, a prototype was fabricated on a 1.6 mm thick FR-4 substrate. The Skyworks SMS7630 Schottky diode was selected as the core rectifying component due to its low turn-on voltage, which makes it suitable for RF energy harvesting applications in the UHF band.

During the experimental evaluation, an SG6-A handheld microwave signal generator (Gaoshun, Shenzhen, Guangdong, China) was employed as the RF excitation source. A conducted measurement configuration was adopted, in which the RF signal was directly injected into the RF input port of the circuit through a coaxial cable under 50 Ω impedance matching conditions, without using an antenna. This configuration was chosen to eliminate uncertainties introduced by wireless propagation effects, such as multipath fading and polarization mismatch. The operating frequency was fixed at 922 MHz. The input power was swept from −19 dBm to 0 dBm with a step size of 1 dBm, and at each power level, data were recorded only after the output voltage reached a steady state. A resistive load was connected to the output of the rectifier, and the output DC voltage was measured using a high-precision digital multimeter. The PCE was then calculated accordingly.

**Figure 15:**
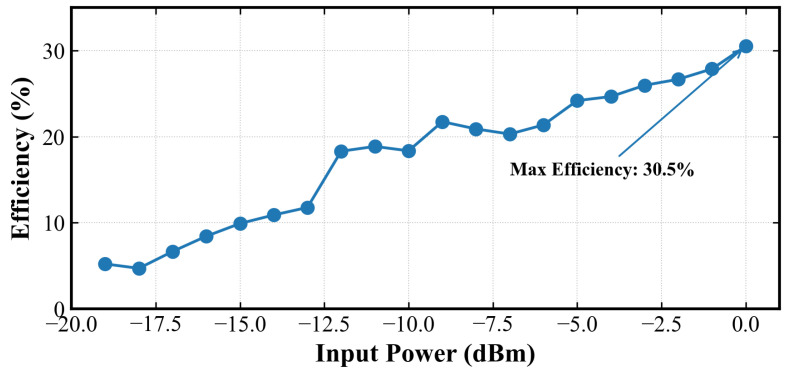
Measured RF-DC power conversion efficiency of the fabricated rectifier circuit versus input power.

Under the above experimental conditions, Figure 15 illustrates the relationship between the PCE and the input power over the range from −19 dBm to 0 dBm. The measured results exhibit a high degree of consistency with the simulation trends, indicating the validity of the proposed circuit model. As the input power increases, the PCE improves significantly, reaching a maximum measured efficiency of approximately 30.5% at 0 dBm. Moreover, even at relatively low input power levels (e.g., −10 dBm), the circuit maintains a comparatively high energy conversion efficiency, demonstrating the effectiveness of the introduced inductive structure and the SMS7630 Schottky diode in reducing reflection losses and threshold-related losses.

#### 3.1.4. Comparison with Recently Reported Energy Harvesting Circuits

To rigorously benchmark the performance of the proposed energy harvesting circuit, this study conducts a comparative analysis against recent reported works, encompassing both CMOS integrated designs and discrete component-based implementations. Table 3 summarizes the critical performance metrics, with a specific focus on operating frequency, fabrication process, peak PCE, as well as implementation complexity and cost. These references represent the latest advancements in the field, including high-performance IC [11,14] and optimized discrete designs [13,15].

As shown in Table 3, the proposed design strategically establishes an optimal engineering balance among efficiency, cost, and signal coverage, rather than focusing solely on maximizing a single metric. Specifically, the synergistic combination of the inductive resonance network and the low-threshold diode boosts the peak PCE from 52.53% in the comparable PCB-based scheme [14] to 68.69%. This substantial increment of approximately 16% validates the efficacy of the improved topology in unlocking the potential of discrete components.

Regarding frequency selection, although reference [12] reports a marginally higher efficiency of 71%, its operation in the 5.8 GHz band incurs a free-space path loss that is approximately 16.2 dB higher than that of the UHF band at equivalent distances. This physical constraint results in severe signal attenuation and a sharply reduced communication range, confirming the superiority of the UHF band for long-range applications. Furthermore, while the peak efficiency of this work trails the 80% achieved by the CMOS process [10], the performance gap of roughly 10% is justifiable given the elimination of high costs associated with photolithography masks and IC tape-out. By delivering near-IC performance using commercial off-the-shelf (COTS) components, the proposed solution demonstrates an ideal equilibrium between manufacturing cost and energy conversion capability.

### 3.2. Load Power Consumption Measurement

To accurately evaluate the power consumption of the proposed system, a dedicated DC measurement setup was established. The system was powered by a WANPTEK GPS305D programmable DC power supply (Shenzhen WanptekElectronic Co., Ltd., Shenzhen, China) with the operating voltage set to 3.1 V. For real-time monitoring, a DigiFaith 6-digit high-precision DC power monitor (DigiFaith, Shenzhen, China) was connected in series between the power supply and the load. Featuring a high measurement resolution of 0.1 μA and 10 μW, this device is capable of capturing subtle power variations. Finally, the voltage and current data were recorded and analyzed via host computer software to eliminate manual reading errors and ensure the reliability of the results.

**Figure 16:**
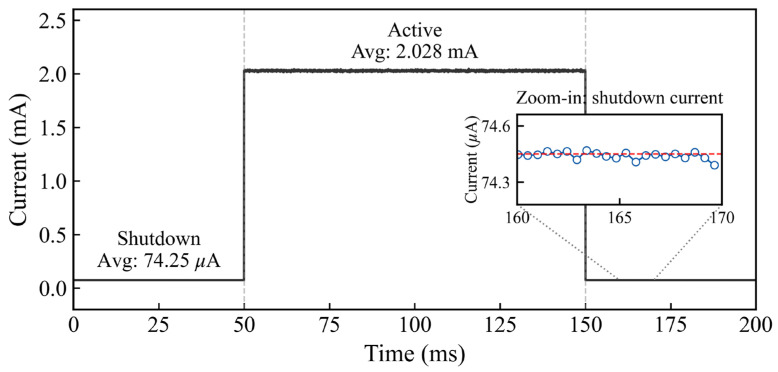
Power consumption profile of the load-MCU-temperature/humidity sensor circuit. The blue circles represent measured current data points. The red dashed line indicates the average value of the measured data, and the grey dashed lines mark the region that is zoomed in and shown in the inset.

As shown in Figure 16, the system demonstrates significant cyclic low-power characteristics. In Shutdown Mode, the current is maintained at approximately 74 μA, a baseline primarily attributed to the quiescent current of the Low Dropout Regulator (LDO) utilized in the current prototype. When the MCU is awakened to drive the SHT10 for measurement, the system generates an operating current of approximately 2.0 mA, with the entire active cycle lasting only about 100 ms. Subsequently, the system cuts off power to peripherals and reverts to sleep mode. Although the current shutdown baseline is limited by the LDO selection, the experimental results fully validate the effectiveness of the proposed intermittent operation strategy in reducing average power consumption.

To further analyze the power distribution, Table 4 details the power consumption of the key system modules. The power consumption of the MCU and LDO was obtained through experimental measurements, whereas the values for the sensor and RFID modules were derived from the typical figures provided in their respective datasheets. It is worth noting that, due to the adopted power gating strategy, the MCU cuts off the power supply to the SHT10 and RFID modules via a GPIO-controlled MOSFET or high-side switch during Shutdown mode. Consequently, the power consumption of these peripherals during the sleep phase is negligible, consisting only of minor leakage currents. The system’s shutdown baseline current of approximately 74 μA is, therefore, primarily composed of the MCU’s Shutdown-mode current and the LDO’s quiescent current.

Overall, this breakdown analysis confirms that the load current remains consistently low during the Shutdown state, effectively reducing the average power consumption of the entire system.

Based on the load power consumption profile presented in Figure 16 and Table 4, the total energy required for a single active cycle lasting 100 milliseconds is calculated to be approximately 0.62 mJ. This specific energy demand serves as the critical threshold that the energy harvesting module must satisfy. To evaluate the impact of this load consumption on the temporal performance of the system, the charging duration required to accumulate 0.62 mJ was analyzed against varying RF input power levels.

**Figure 17:**
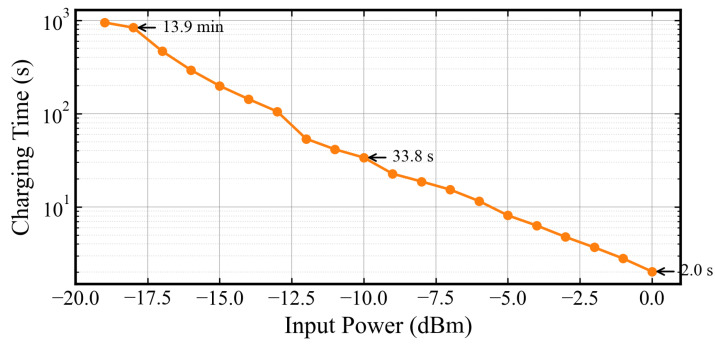
Calculated capacitor charging duration required to replenish the single-cycle energy consumption under different input power.

As shown in Figure 17, the low energy demand of the optimized load enables rapid recharge cycles. The required charging duration decreases sharply as the input power rises, which necessitates a logarithmic scale representation to visualize the wide dynamic range. Under strong signal conditions of 0 dBm, the system needs only about 2.0 s to replenish the measured 0.62 mJ energy, thereby facilitating near real-time monitoring. In contrast, the charging period extends to approximately 13.9 min in weak signal scenarios at −19 dBm. This analysis confirms that the proposed low-power management strategy effectively minimizes the energy accumulation latency and ensures a practical operation frequency even under constrained RF power conditions.

### 3.3. Performance Evaluation of Anti-Collision Algorithms

**Figure 18:**
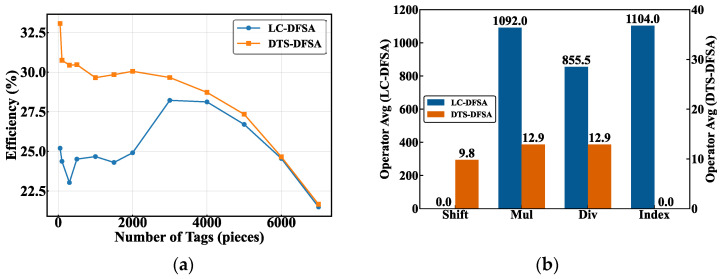
Performance comparison between LC-DFSA and DTS-DFSA algorithms under varying tag populations: (**a**) identification efficiency versus the number of tags; (**b**) operator-count distribution at a fixed tag population of 100.

As illustrated in Figure 18a, a significant discrepancy in identification efficiency occurs between the LC-DFSA algorithm and the proposed DTS-DFSA algorithm. Under the scenarios with a small number of tags, the DTS-DFSA algorithm achieves a peak identification efficiency of approximately 33%, and its performance consistently outperforms that of the LC-DFSA algorithm under most operating conditions. Although the efficiency gap between the two types of algorithms exhibits a gradual narrowing trend as the number of tags increases, the DTS-DFSA algorithm maintains higher stability across the entire range of tag quantities.

Further analysis of computational complexity, as shown in Figure 18b, reveals that compared with the LC-DFSA algorithm, the DTS-DFSA algorithm requires significantly fewer multiplication, division, and indexing operations. Its frame length adjustment and collision resolution processes primarily rely on lightweight shift operations. This simplification of arithmetic operations directly enables an effective reduction in algorithm complexity.

To further elucidate the core advantages of the proposed DTS-DFSA, Table 5 presents an in-depth quantitative comparison between the proposed algorithm and state-of-the-art techniques in the anti-collision domain. These techniques include the deep learning-based D-G-MFSA [11], the physical layer separation-based CS-OMP [12], and the deterministic tree protocol-based DRCT [13].

First, regarding the efficiency-cost trade-off, although DRCT and D-G-MFSA achieve remarkable channel utilization and theoretical throughput rates of 84.8% and 36.8%, respectively, these performance gains are attained at the expense of exponentially increasing computational costs. As evidenced by the instruction-level operator statistics in Figure 18b, the intensive neural network inference or matrix iterative logic mandated by these high-performance algorithms typically incurs clock cycles and memory overheads that are one to two orders of magnitude higher than those of the proposed scheme.

Second, in terms of low-level implementation, DTS-DFSA demonstrates superior engineering adaptability. In contrast to existing solutions that rely heavily on Floating-Point Units or complex recursive management, the proposed scheme ingeniously simplifies the frame length adjustment logic into low-level stepped bit-shifting. This achieves a “zero-multiplication and zero-division” paradigm at the instruction set execution level. For low-power RFID sensing platforms that lack hardware floating-point support and are severely resource-constrained, this extreme logical simplification translates into reduced response latency and extended node operational lifespan.

Finally, concerning dynamic robustness, DTS-DFSA eliminates the dependency on prior knowledge of the initial tag population. Unlike THDFSA, which is highly sensitive to estimation errors, the proposed scheme can rapidly converge to the optimal frame length solely through real-time collision rate feedback. In summary, DTS-DFSA significantly compresses the computational footprint while maintaining a competitive efficiency of 33.0%. This Pareto balance, specifically tailored for resource-constrained environments, constitutes the distinct engineering competitive advantage of the proposed solution over state-of-the-art high-performance algorithms.

## 4. Conclusions

Aiming at the key issues of low RF energy harvesting efficiency, high sensor power consumption, and high complexity of tag anti-collision algorithms in RFID sensor systems, this paper proposes a hardware–software co-optimization scheme for passive RFID sensing systems.

To improve RF energy harvesting efficiency, the effects of introducing an input inductor and adopting the low-threshold RF Schottky diode SMS7630 are analyzed and validated through theoretical and simulation studies. For reducing sensor power consumption, the system design focuses on three aspects: MCU low-power mode, operation scheduling strategy, and power control of the SHT10 sensor, all verified through physical experiments. Regarding anti-collision optimization, the interaction mechanism between the reader and tags in frame-slotted algorithms is reviewed, and a DTS-DFSA algorithm balancing complexity and identification efficiency is proposed and simulated in software.

The results show that the improved RF energy harvesting circuit, with the added inductor, low-pass filter, and diode replacement, achieves significantly higher energy conversion efficiency and smoother output waveforms. In the standby state, the overall load current controlled by the MCU decreases notably. The proposed DTS-DFSA algorithm demonstrates improved computational simplicity and higher identification efficiency.

In conclusion, the proposed RFID sensor system, integrating the optimized RF energy harvesting circuit and anti-collision algorithm, achieves effective improvements in energy harvesting efficiency, power consumption control, and anti-collision performance. However, further optimization is still possible: the energy harvesting circuit could employ integrated implementations to reduce diode threshold losses; lower-power MCUs and sensors could be adopted to further minimize system consumption; and under small tag populations (e.g., 50 tags), the DTS-DFSA algorithm exhibits reduced stability, with identification efficiency occasionally falling below that of LC-DFSA, indicating the need for further refinement.

## Figures and Tables

**Table 1:** Key parameters of several commonly used RF diodes considered in the rectifier design.

Model	*V_f_*(mV) @ 1 mA	*I_r_* @ 2V(nA)	C_t_(pF) @ 0.15 V
HSMS285C	250	175	0.30
HSMS282x	340	100	1.00
HSMS-2860 [17]	250–350	−	0.3
BAT17-04W	340	250	0.61
SMS7621	260–320	−	0.25
SMS7630 [18]	135–240	−	0.3

**Table 2:** Frame length adjustment strategies based on *CR*.

Collision Probability	Adjustment	Expression	Behavior
High (CR>0.7)	Double frame length	$N_{next}$ = N≫1	Increase the frame length
Low (CR<0.3)	Halve frame length	$N_{next}$ = N≪1	Shorten the frame length
Stable (0.3 <CR<0.7)	Maintain frame length	$N_{next}$ = N	Maintain the frame length

**Table 3:** Performance comparison between the proposed design and recently reported energy harvesting circuits.

Ref.	Technology	Frequency	Peak PCE	Complexity
Zheng [7]	CMOS (0.18 µm)	UHF	70–80%	High
Sidibe [8]	CMOS (Fully Integrated)	900 MHz	31.77%	High
Kumar [9]	Discrete (Plug-in)	5.8 GHz	71%	Moderate
Liu [10]	Discrete (PCB)	2.4 GHz	52.53%	Low
This Work	Discrete (SMS7630)	920–925 MHz	68.69%	Low

**Table 4:** Measured and Estimated Power Consumption of Key Modules.

Module	Model	Active Current (mA)	Sleep Current (μA)
MCU	STM32L431 ^1^	1.0300	3.15
LDO	ME6211C33M5G ^2^	0.0711	71.10
Sensor	SHT10	1.1000	0.0
RFID Tag	EM4325	0.0015	0.0
Total	-	2.2025	74.25

^1^ STM32L431 (STMicroelectronics, Plan-les-Ouates, Switzerland). ^2^ ME6211C33M5G LDO (Nanjing Micro One Electronics Inc., Nanjing, Jiangsu, China).

**Table 5:** Performance and structural characteristics comparison of anti-collision algorithms.

Algorithm	Adjustment Mechanism	Identification Efficiency	Key Operations
D-G-MFSA	LSTM Prediction	36.8%	Matrix Mul., Float Ops.
CS-OMP	CS Reconstruction	Moderate	Matrix Iteration, Variance
DRCT	Dual Response Tree	84.8%	Recursion, Prefix Match
LC-DFSA [15]	Discrete LUT	28.2%	Logic Ops., LUT Access
THDFSA	Two-Threshold Policy	35–38%	Threshold Compare, Frame Update
DTS-DFSA	Dual-Threshold CR	33.0%	Bit-Shifting, Addition

## Data Availability

The raw data supporting the conclusions of this article will be made available by the corresponding author on request.
